# Police Pursuit Fatalities in the US, 2009 to 2023

**DOI:** 10.1001/jamanetworkopen.2026.4340

**Published:** 2026-04-01

**Authors:** Andrew Hendrix, Tamriage Martin, Joshua Gazzetta, Jade Nunez

**Affiliations:** 1 Department of General Surgery, University of Virginia, Charlottesville

## Abstract

**Importance:**

Police pursuits account for approximately 1% of motor vehicle fatalities and expose suspects, officers, and bystanders to preventable harm. Contemporary population-based estimates of fatality trends and context-specific risk remain limited.

**Objective:**

To evaluate the characteristics of and trends in fatal vehicle crashes involving police pursuit in the US between 2009 and 2023 and the factors associated with these crashes.

**Design, Setting, and Participants:**

This cross-sectional study used National Highway Traffic Safety Administration Fatality Analysis Reporting System (FARS) data between January 2009 and December 2023. These nationwide, population-based data included individuals involved in fatal motor vehicle crashes involving police pursuits. The data analysis was performed between August 2025 and February 2026.

**Exposures:**

Police motor vehicle pursuits, as documented in the FARS database.

**Main Outcomes and Measures:**

The primary outcomes were pursuit-related motor vehicle crash fatalities per year and associated factors, including urban vs rural settings, interstate vs noninterstate roads, US census regions, time of day, and day of the week. A negative binomial regression was used to model fatality rates controlling for population and crash frequency.

**Results:**

Between 2009 and 2023, there were 5425 fatal police pursuit-related crashes involving 8307 vehicles and 14 497 persons, resulting in 6352 deaths (mean [SD], 423 [84] deaths per year). Fatal crashes occurred most often in urban settings (3069 [57%]), at night (3794 [70%]), on noninterstate roads (4825 [89%]), and with documented speeding (4183 [77%]). Nonmotor vehicle occupants represented 270 fatalities (4%). When controlling for population and crashes, fatalities increased by 2% (95% CI, 1%-3%; *P* < .001) annually. Compared with the Northeast, fatalities were significantly higher in the South (difference, 336% [95% CI, 284%-395%]; *P* < .001), the Midwest (difference, 110% [95% CI, 84%-140%]; *P* < .001), and the West (difference, 95% [95% CI, 70%-123%]; *P* < .001).

**Conclusions and Relevance:**

In this cross-sectional study of the FARS database from 2009 to 2023, police pursuit-related fatalities increased over 15 years. Higher rates of fatal crashes were concentrated in identifiable settings including nighttime hours, urban corridors, noninterstate roadways, and southern states. The findings support risk-based restrictions, mandatory national pursuit reporting, and investment in nonpursuit alternatives among US police.

## Key Points

**Question:**

What were the characteristics of and trends in fatal motor vehicle crashes involving police pursuits in the US between 2009 and 2023, and were any factors associated with these crashes?

**Findings:**

In this cross-sectional study of 5425 fatal pursuit crashes over 15 years, deaths increased 2% annually after adjusting for population and crash frequency. More fatal pursuit crashes occurred at night, in urban areas, on noninterstate roads, and in southern states.

**Meaning:**

The findings support risk-based restrictions, mandatory national reporting, and investment in nonpursuit technologies among US police.

## Teaser

This cross-sectional study evaluates the epidemiology, risk factors, and temporal trends associated with fatal vehicle crashes involving police pursuit in the US between 2009 and 2023.

## Introduction

Police pursuits are a hazardous practice, with risks that have grown amid increasing automobile traffic, greater pedestrian activity, and widespread use of mopeds, electric bikes, and scooters.^1,2,3^ National surveys document approximately 68 000 pursuits annually, accounting for 1% of all motor vehicle fatalities.^4,5^ Most begin with a traffic violation,^6^ often escalating into high-speed crashes causing serious injuries and death.^4,7^ Yet, public surveys spanning 3 decades have shown consistent public support for pursuits following serious crimes but strong opposition for pursuits following minor offenses or in high-risk conditions.^8,9,10^ Pursuits expose suspects, officers, and uninvolved bystanders to harm and represent a preventable source of public risk.^4,8,11^ These crashes create severe multiple trauma requiring complex, costly emergency care,^12,13,14,15,16^ placing undue burden on communities and trauma systems.

National recommendations, published in 2023 by the Police Executive Research Forum,^17^ aimed to improve pursuit policy, but national evidence integrating population burden with context-specific risk remains scarce. Existing studies are dated,^11^ are geographically narrow, or compile data from nonstandardized sources without key variables, such as urbanicity, roadway class, time of day, or alcohol involvement, within adjusted models.^18^ The recent national analysis by Bather and Goodman^19^ using a journalist-compiled database reported an increasing number of deaths but was limited by inconsistent definitions and a short time series. No prior studies, to our knowledge, have used nationally representative surveillance systems to calculate population-based fatality rates over time with consistent definitions and contextual adjustment.

We used the Fatality Analysis Reporting System (FARS), a National Highway Traffic Safety Administration (NHTSA) database,^20^ from 2009 to 2023, to test the hypothesis that pursuit-related fatalities have increased as at-risk road user presence has grown. We estimated contemporary, population-based rates of crash-related fatalities involving police pursuits at national and regional levels, temporal trends; and adjusted rates across key contexts including urbanicity, roadway type, time of day, and geographic region. Using consistent definitions and a transparent classification of pursuit involvement and decedent roles, we aimed to identify where and when fatal risk was concentrated to inform prevention and planning.

## Methods

### Data Source

We performed a cross-sectional study by accessing, collating, and analyzing the FARS database from January 2009 to December 2023. After the study proposal was deemed nonhuman participant research by the University of Virginia institutional review board in accordance with the Code of Federal Regulations (45 CFR §46.102).^21^ Supplemental queries into the FARS database were used for supplemental data and confirmation of raw data. The results of this study were reported in accordance with the Strengthening the Reporting of Observational Studies in Epidemiology (STROBE) guideline for cross-sectional studies.

FARS is a publicly available nationwide database that details yearly data regarding fatalities related to motor vehicle crashes within 30 days. FARS draws on multiple sources of information provided by individual states including police crash reports, death certificates, coroner or medical examiner reports, emergency medical service reports, and state highway department data. FARS then collates the information and codes for persons and vehicles per crash.^22^ Persons are split into 2 categories based on motor vehicle occupancy at the time of the crash. Motor vehicle occupants can be the driver of the motor vehicle in transport, the passenger of the motor vehicle in transport, an occupant of a motor vehicle not in transport, or an unknown occupant. Nonmotor vehicle occupants can be occupants of a nonmotor vehicle transport device, a pedestrian, a bicyclist, a pedalcyclist, a person on a personal conveyance, a person in or on a building, or an unknown type of nonmotorist. FARS does not measure the total number of individuals or vehicles encountered during a pursuit prior to the terminating crash. Individuals encoded include only those who are reported at the site of the terminating crash.

Continuous variables obtained from the FARS included year and fatality counts. Categorical variables included the state; interstate status (roadway designated by the Federal Highway Administration with at least 4 lanes); rural or urban (Federal Highway Administration–approved adjusted census boundaries) designation; pedestrian, pedalcyclist, and/or speeding involvement (documented by police officer); time of day (6:00 am to 5:59 pm or 6:00 pm to 5:59 am); and police pursuit involvement. The NHTSA provides national statistics on crashes that were used in the data analysis. The US Census Bureau^23,24^ provides yearly national and regional estimates of regional and state population characteristics that were used in the data analysis.

### Statistical Analysis

The data analysis was performed between August 2025 and February 2026. Characteristics of fatal motor vehicle crashes involving police pursuits were described as frequency and percentage for categorical variables. Mean and SD were used to describe normally distributed continuous variables, with use of median and IQR to describe nonnormally distributed continuous variables. A negative binomial regression was used to analyze motor vehicle pursuit fatality rates with fatality count as the response variable. Variables included year, census region, rural or urban setting, interstate or noninterstate designation, day or nighttime, weekday or weekend, and documented speeding. Incomplete data, such as unknown time-of-day status, was coded as unknown for categorical variables and included in the final model to assist with explaining overall variance in the data. To analyze trends controlling for crash frequency and population growth, both yearly population, as reported by the US Census Bureau,^23^ and yearly motor vehicle crashes, as reported by the NHTSA,^25^ were included as offset variables. A Poisson regression model was initially considered, however preliminary analysis demonstrated evidence of overdispersion, and therefore a negative binomial regression was deemed more appropriate. To evaluate for collinearity among variables, we examined generalized variance inflation factors ([GVIF]^1/[2 ×^ *^df^*^]^) and found all values below 1.2 to be indicative of no significant collinearity. To minimize the risk of a type 1 error, we performed the Benjamini-Hochberg false discovery rate procedure on the 2-tailed significance threshold of *P* = .05.^26^ After adjustment, no variables lost their significance, and therefore unadjusted *P* values were reported. Raw coefficients from negative binomial regression are exponentiated (e*^x^*) to calculate incident rate ratios (IRRs) (eTable 1 in Supplement 1). IRRs of variables are reported as percentage change using the formula (1 − IRR) × 100. Statistical analysis and data visualization were performed using R, version 4.5.1^27^ via RStudio^28^ (R Project for Statistical Computing).

## Results

**Figure 1.**
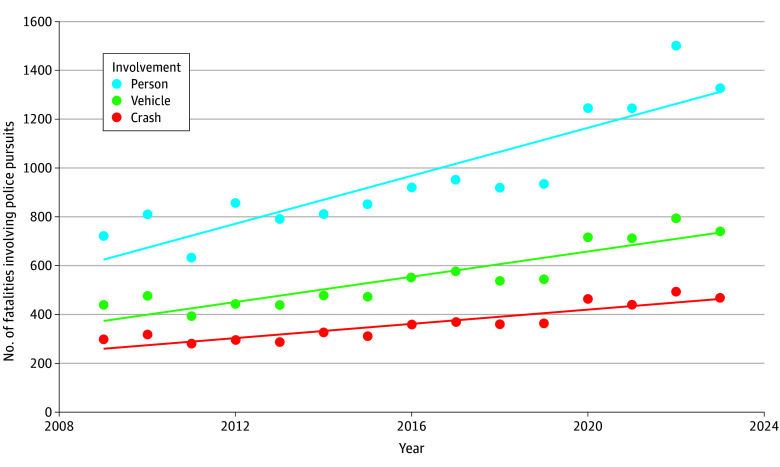
Line Graph of Trends of Fatal Police Pursuit Crashes, Vehicles, and Persons Involved

**Figure 2.**
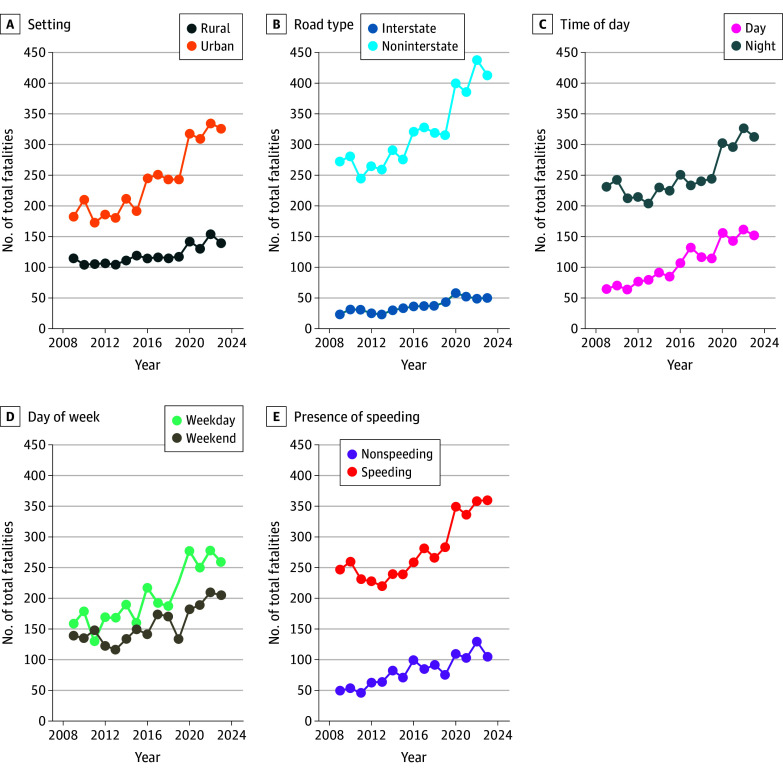
Line Graph of Trends in Yearly Fatalities by Category

Between 2009 and 2023, there were a total of 5425 fatal motor vehicle crashes involving police pursuits (mean [SD], 362 [69] fatal crashes per year). The number of crashes varied between the minimum 280 in 2011 and the maximum 496 in 2022 (Figure 1). The 5425 fatal motor vehicle crashes involved a total of 8307 vehicles (mean [SD], 554 [123] vehicles per year) and 14 497 persons (mean [SD], 936 [238] persons per year). In total, fatal police pursuits resulted in 6352 persons killed (mean [SD] 423 [84] deaths per year). Of the total persons killed, 6063 (95%) were occupants (mean [SD], 404 [79] occupants per year), 270 (4%) were nonoccupants (mean [SD], 18 [6] nonoccupants per year), and 19 (3%) were unknown. Over the 15-year time period, 350 total nonoccupants were involved in fatal police pursuits, with 270 pursuits (77%) resulting in fatalities in contrast to 14 050 occupants involved in fatal police pursuits with 6042 fatalities (43%). Fatal police pursuits occurred more often in urban settings (3069 [57%]) compared with rural settings (1801 [33%]), more often at nighttime (3794 [70%]) than daytime (1627 [30%]), and less commonly on interstate roads (564 [10%]) compared with noninterstate roads (4825 [89%]) and frequently involved documented speeding (4183 [77%]), as detailed in Figure 2. The IRR for fatality for urban settings was 1.75 (95% CI, 1.62-1.89) compared with rural settings, and the IRR for fatality for nighttime hours was 1.94 (95% CI, 1.80-2.10) compared with daytime hours (eTable 1 in Supplement 1).

**Figure 3.**
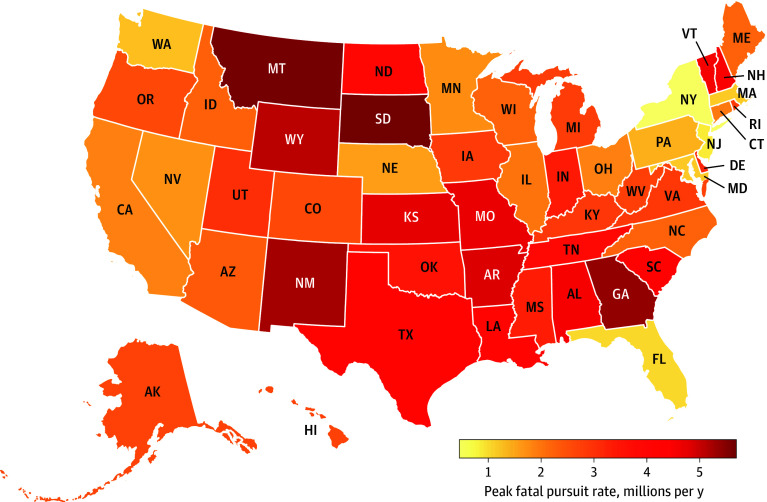
Map of Peak Yearly Fatal Pursuits Per Capita in US States, 2009 to 2023

States varied widely in their maximum yearly number of fatalities per capita associated with fatal police pursuits (Figure 3 and eTable 2 in Supplement 1). The South region sustained the most fatalities associated with fatal police pursuits (n = 3191) compared with the Northeast, which had the least (n = 498). After accounting for regional differences of population, the Northeast had the least mean (SD) fatalities per million per year (0.57 [4.03]) compared with the South (1.60 [16.80]), the Midwest (1.35 [6.55]), and the West (1.04 [12.16]) (Table). When controlling for year, roadway type, time of day, and other factors, compared with the Northeast, the IRR for fatality in the South was 4.36 (95% CI, 3.84-4.95) after accounting for population and baseline crash rates (eTable 1 in Supplement 1).

**Table.** Summary of Fatality Counts Per Year by US Census Region, 2009 to 2023^a^

Region	Fatalities, No.		
	Total	Per million per year, mean (SD)^b^	State level, median (IQR) (range)
Midwest	1410	1.35 (6.55)	7 (2-12) [0-29]
Northeast	498	0.57 (4.03)	2 (1-6) [0-18]
South	3191	1.60 (16.80)	8 (3-15) [0-128]
West	1253	1.04 (12.16)	2 (1-5) [0-73]

^a^ Data source: Fatality Analysis Reporting System. Population data sourced from the US Census Bureau’s 2023 regional estimates.^23^

^b^ Calculated as (Total Fatalities/15 years/2023 Census Population) × 1 000 000.

**Figure 4.**
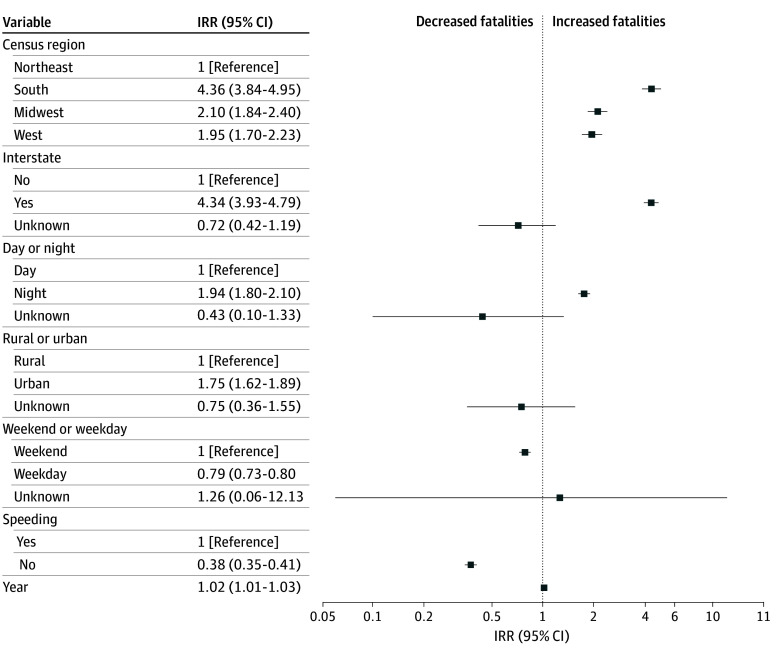
Forest Plot of Incident Rate Ratios of Police Pursuit Crash Fatalities IRR indicates incident rate ratio.

The final negative binomial model had a residual fit of 1020 on 1120 *df* with a theta value of 6.66 indicating a good overall fit (eFigure in Supplement 1). The model’s Akaike information criterion was 5110.5 compared with the preliminary Poisson model (Akaike information criterion = 5473.2). When controlling for other variables, there was a significant positive yearly increase in fatalities of 2% (95% CI, 1%-3%; *P* < .001) (Figure 4). The Midwest (110% [95% CI, 84%-140%]; *P* < .001), the South (336% [95% CI, 284%-395%]; *P* < .001), and the West (95% [95% CI, 70%-123%]; *P* < .001) all had significantly higher fatalities compared with the Northeast. There was a significant increase in fatalities in urban settings (75% [95% CI, 62%-89%]; *P* < .001) compared with rural settings and a significant increase on noninterstate roads (334% [95% CI, 293%-379%]; *P* < .001) compared with interstate roads. Fatalities were higher at nighttime compared with daytime (94% [95% CI, 80%-110%]; *P* < .001) and were significantly lower when speeding was not documented (−62% [95% CI, −65% to −59%]; *P* < .001) and on the weekends compared with weekdays (−21% [95% CI, −27% to −15%]; *P* < .001). There were no statistically significant changes in unknown fatalities for rural and urban settings (−25% [95% CI, −64% to 55%]; *P* = .37), interstate roads (−28% [95% CI, −58% to 19%]; *P* = .18), and weekdays and weekends (−26% [95% CI, −94% to 113%]; *P* = .85).

## Discussion

In this cross-sectional study, pursuit-related motor vehicle fatalities increased over time and clustered in identifiable contexts. Fatalities were more common at night, on noninterstate roadways, and in urban settings. Burden varied across regions, with persistently higher rates in several southern states. Averaged across the study period per day, there was approximately 1 fatal motor vehicle crash (362 per year), with almost 3 individuals involved (936 per year), and 1 individual killed (423 per year) due to a police pursuit.

Prior work has established that police pursuits are associated with substantial mortality risk and frequently result in crashes and injuries.^5,11,19^ Estimates indicate that approximately 40% of pursuits culminate in a crash, and about 20% result in injuries.^4,29^ Early surveillance using the FARS (1982 to 2004) reported a mean 323 pursuit-related fatalities annually, with elevated risk in urban settings.^11^ The US Department of Justice later reported 351 pursuit-related deaths in 2012 based on the Law Enforcement Management and Administrative Statistics survey, although this figure reflected only a single year and lacked adjustment for population growth or crash frequency.^5^ Most recently, Bather and Goodman^19^ examined a journalist-compiled database (2017 to 2021) and found a rising trend and geographic disparities, particularly in southern states.^30,31^ Our study extends this work in 3 ways. First, we evaluated recent, continuous surveillance, allowing assessment of both short-term fluctuations and sustained trends. Second, we examined population-based rates that account for both demographic growth and the changing denominator of all motor vehicle crashes, revealing a 2% annual increase in an adjusted fatality rate even as overall traffic deaths have recently stabilized. Third, we analyzed a standardized federal surveillance system with uniform definitions and comprehensive geographic coverage, eliminating concerns about case ascertainment bias that can affect compilations from heterogeneous news sources or single-year surveys.

Studies have identified urban roads, nighttime hours, and southern states as high-risk settings for pursuit fatalities. Urban roadways have greater police presence, higher citation rates, more traffic congestion, and denser pedestrian activity.^32,33^ Nighttime pursuits involve distinct hazards including prolonged visual reaction times under low-visibility conditions; increased alcohol involvement; and night-shift fatigue linked to excessive sleepiness, near-crashes, and errors among police.^34,35,36^ Regional variation in southern states reflects higher average speeds, lower safety-belt use, and more vehicle occupants.^37^ Our analysis quantified these risks within a single adjusted framework. When controlling for year, roadway type, time of day, and other factors, southern states showed more than 4 times higher fatality risk than in Northeast states after accounting for population and baseline crash rates. Urban settings and nighttime hours were associated with approximately doubled rates independent of other variables. These adjusted estimates isolate where higher rates concentrate after accounting for confounders, moving beyond descriptive counts to comparative measures that target interventions.

Our findings have implications for multiple stakeholders. For policymakers, the results support risk-based restrictions on ground pursuits, particularly for minor offenses and in high-risk conditions such as night driving, urban corridors, and noninterstate arterials. National surveys show pursuit rates drop from 17 per 100 officers under discretionary policies to 8 to 9 per 100 officers under restrictive policies.^5^ Standardized national pursuit reporting would enable evaluation of whether policy changes reduce fatalities. For law enforcement agencies, alternatives to ground pursuit merit serious consideration. Global Positioning System tagging^38^ and drones are options for these high-burden contexts.^39,40,41^ Field trials of Global Positioning System tagging systems achieved 80% apprehension rates with no injuries or property damage,^38,42,43^ whereas pursuit crashes average $8500 to $35 000 in economic losses per event,^44,45,46,47,48,49,50^ making technology adoption cost-effective. For trauma systems and practitioners, the temporal and geographic clustering of fatalities indicates where Emergency Medical Services coverage, transfer protocols, and operative capacity should be aligned with demand. Mandatory national pursuit reporting would enable evaluation of whether policy adoption reduces the 27% to 35% of deaths borne by uninvolved road users.^4^ Future work should link fatal-crash surveillance with pursuit logs and policy datasets to test whether adoption of risk-based policies and technology reduces fatalities.

### Limitations

This study has several limitations. The FARS likely underestimates the total burden, as it relies on police crash reports, in which pursuit involvement may be underreported or misclassified.^51^ Additionally, because the FARS captures data only on the terminal crash rather than the full duration of a police pursuit, measures of people involved and nonoccupants involved are restricted to those associated with the final crash event, do not represent the total number of individuals encountered during the pursuit, and are likely underestimates of the broader population exposed to pursuit-related risk. The 30-day mortality window excludes deaths after prolonged hospitalization and captures no nonfatal injury data, which also may contribute to the aforementioned underestimation. Deidentified records prevented complete role classification, limiting granular assessment of outcomes for suspects, officers, and bystanders. This also limited the assessment of individual-level factors like race or neighborhood socioeconomic factors. Prior research has demonstrated substantial variation in policing exposure and police violence by race, location, and neighborhood characteristics.^52,53,54,55,56,57^ Specifically, Black and Hispanic individuals experience disproportionately higher rates of police stops, use of force, and fatal encounters, particularly in urban, socioeconomically disadvantaged communities, and in the Southwest and Western regions of the US.^52,53,54,55,56,57^ Additionally, despite clustering by state, residual confounding from unmeasured factors remains possible. These constraints are offset by the FARS being the only standardized federal surveillance system, to our knowledge, with consistent definitions and comprehensive coverage, the 15-year timeframe to reduce the impact of random variation, and the multivariable framework to adjust for key contextual factors.

## Conclusions

In this cross-sectional study of the FARS database from 2009 to 2023, police pursuit-related motor vehicle fatalities increased over 15 years. Higher rates concentrated in identifiable settings: nighttime hours, urban corridors, noninterstate roadways, and southern states. Findings support risk-based restrictions, mandatory national pursuit reporting, and investment in nonpursuit alternatives among US police.

## Notes

Supplement 1. **eTable 1.** Incident Rate Ratios of Negative Binomial Regression **eTable 2.** Peak Yearly Fatalities From Police Pursuits by State **eFigure.** Predicted and Actual Fatalities by Region

Supplement 2.
Data Sharing Statement
