# Supplementary material for: Police Pursuit Fatalities in the US, 2009 to 2023
Source: JAMA Netw Open. 2026 Apr 1;9(4):e264340. doi: 10.1001/jamanetworkopen.2026.4340 (PMC13044674; doi:10.1001/jamanetworkopen.2026.4340)
Supplement: Supplement 1. — eTable 1. Incident Rate Ratios of Negative Binomial Regression eTable 2. Peak Yearly Fatalities From Police Pursuits by State eFigure. Predicted and Actual Fatalities by Region [file jamanetwopen-e264340-s001.pdf]

Supplemental Online Content

Hendrix A, Martin T, Gazzetta J, Nunez J. Police pursuit fatalities in the US, 2009 to 2023. *JAMA Netw Open*. 2026;9(4):e264340. doi:10.1001/jamanetworkopen.2026.4340

eTable 1. Incident Rate Ratios of Negative Binomial Regression
eTable 2. Peak Yearly Fatalities From Police Pursuits by State
eFigure. Predicted and Actual Fatalities by Region

This supplemental material has been provided by the authors to give readers additional information about their work.

© 2026 Hendrix A et al. *JAMA Network Open*.

eTable 1. Incident Rate Ratios of Negative Binomial Regression

Predictor	IRR (95% CI)	P Value	Adjusted P Value[a]
Year	1.02 (1.01-1.03)	<.001	<.001
Census Region (Ref: Northeast)			
Midwest	2.10 (1.84-2.40)	<.001	<.001
South	4.36 (3.84-4.95)	<.001	<.001
West	1.95 (1.70-2.23)	<.001	<.001
Location (Ref: Rural)			
Urban	1.75 (1.62-1.89)	<.001	<.001
Unknown	.75 (.36-1.55)	0.37	0.40
Roadway Type (Ref: Interstate)			
Non-Interstate	4.34 (3.93-4.79)	<.001	<.001
Unknown	.72 (.42-1.19)	0.18	0.22
Time of Day (Ref: Day)			
Night	1.94 (1.80-2.10)	<.001	<.001
Unknown	.43 (.10-1.33)	0.19	0.22
Speeding (Ref: Yes)			
No	.38 (.35-.41)	<.001	<.001
Timing (Ref: Weekday)			
Weekend	.79 (.73-.80)	<.001	<.001
Unknown	1.26 (.06-12.13)	0.85	0.85

[a]To account for multiple tests the Benjamini-Hochberg False Discovery Rate (FDR) procedure on our two-tailed significance threshold of p=.05.

© 2026 Hendrix A et al. *JAMA Network Open*.

eTable 2. Peak Yearly Fatalities From Police Pursuits by State

State	Peak Year	Rate per 1M	Fatalities, No.	State	Peak Year	Rate per 1M	Fatalities, No.
South Dakota	2018	5.69	5	Iowa	2016	2.87	9
Montana	2018	5.65	6	Rhode Island	2012	2.84	3
Georgia	2020	5.40	58	Michigan	2014	2.82	28
New Mexico	2017	5.26	11	Hawaii	2016	2.80	4
Wyoming	2015	5.12	3	West Virginia	2019	2.79	5
Arkansas	2023	4.89	15	Alaska	2012	2.74	2
Kansas	2018	4.81	14	Colorado	2018	2.63	15
Missouri	2018	4.73	29	Oregon	2010	2.61	10
Vermont	2023	4.62	3	Arizona	2020	2.37	17
Alabama	2020	4.57	23	Idaho	2018	2.28	4
New Hampshire	2015	4.49	6	Maine	2011	2.26	3
South Carolina	2023	4.45	24	North Carolina	2022	2.24	24
Texas	2022	4.25	128	Wisconsin	2018	2.24	13
Louisiana	2022	4.14	19	Illinois	2020	2.03	26
North Dakota	2016	3.97	3	Connecticut	2017	1.96	7
Tennessee	2021	3.88	27	California	2021	1.86	73
Oklahoma	2017	3.56	14	Ohio	2023	1.86	22
Indiana	2021	3.37	23	Minnesota	2018	1.78	10
Mississippi	2013	3.34	10	Nevada	2016	1.71	5
Delaware	2014	3.21	3	Nebraska	2016	1.57	3
Utah	2014	3.06	9	Pennsylvania	2011	1.41	18
District of Columbia	2022	2.96	2	Washington	2013	1.29	9
Kentucky	2018	2.91	13	Maryland	2012	1.19	7
Virginia	2021	2.89	25	Massachusetts	2019	1.16	8
				Florida	2010	1.06	20
				New Jersey	2017	0.90	8
				New York	2016	0.46	9

© 2026 Hendrix A et al. *JAMA Network Open*.

eFigure. Predicted and Actual Fatalities by Region

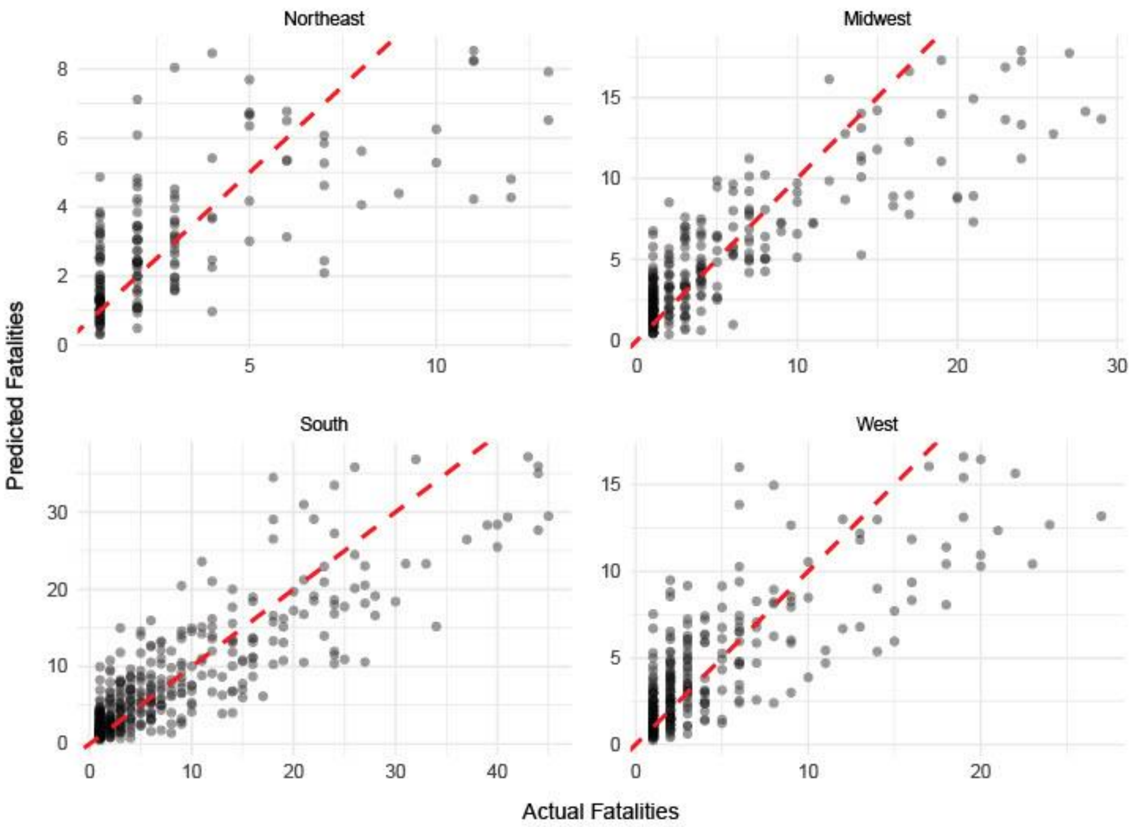

© 2026 Hendrix A et al. *JAMA Network Open*.
