# Supplementary material for: Police Pursuit Fatalities in the US, 2009 to 2023
Source: JAMA Netw Open. 2026 Apr 1;9(4):e264340. doi: 10.1001/jamanetworkopen.2026.4340 (PMC13044674; doi:10.1001/jamanetworkopen.2026.4340)
Supplement: Supplement 2. — Data Sharing Statement [file jamanetwopen-e264340-s002.pdf]

Data Sharing Statement

Hendrix. Police Pursuit Fatalities in the US, 2009 to 2023. *JAMA Netw Open*. Published April 01, 2026. doi:10.1001/jamanetworkopen.2026.4340

Data
Data available: No

Additional Information
Explanation for why data not available: already publicly available in FARS database
